# Off-target anti-leukemic effects of antibiotics: mechanisms and therapeutic insights

**DOI:** 10.1007/s00280-026-04916-7

**Published:** 2026-07-06

**Authors:** Jason Charamis, Nikolaos Katzilakis, Eftichia Stiakaki, Ioannis Kyriakidis

**Affiliations:** 1 Center of Clinical, Experimental Surgery and Translational Research, Biomedical Research Foundation, Academy of Athens, Athens, 11527 Greece; 2 Department of Pediatric Hematology-Oncology & Autologous Hematopoietic Stem Cell Transplantation Unit, University Hospital of Heraklion & Laboratory of Blood Diseases and Childhood Cancer Biology, School of Medicine, University of Crete, Heraklion, 71003 Greece; 3 Section of Biochemistry, Molecular Biology, Cellular and Developmental Biology, Department of Biology, University of Crete, Heraklion, 70013 Greece

**Keywords:** Antibiotics, Drug interactions, Drug side effect, Hematologic toxicity, Ribosome, Leukemia

## Abstract

Antibiotics are among the transformative advances in medicine, but many interact with mammalian cellular targets and pathways beyond their antimicrobial activity. A clinically important expression of these off-target effects is hematologic toxicity, including immune-mediated cytopenias and direct bone marrow suppression. This narrative review examines whether the same biology that injures normal hematopoietic cells can, in selected contexts, reveal therapeutically exploitable vulnerabilities in leukemia. We synthesize molecular, clinical, and preclinical evidence and organize it into an integrative framework linking mitochondrial translation inhibition, mitonuclear imbalance, oxidative phosphorylation failure, reactive oxygen species generation, DNA/topoisomerase stress, autophagy and lysosomal-flux blockade, and apoptosis modulation with both hematotoxicity and antileukemic activity. The strongest preclinical evidence supports selected tetracyclines, macrolides, and oxazolidinones, whereas evidence for beta-lactams, glycopeptides, polymyxins, rifamycins, fluoroquinolones, and folate-pathway agents remains more limited or largely hypothesis-generating. Importantly, antibiotic-induced cytopenia should not be interpreted as proof of leukemia selectivity: immune-mediated toxicity, supratherapeutic in vitro exposure, normal progenitor injury, pharmacokinetic constraints, microbiome effects, and resistance mechanisms all narrow the translational window. Overall, antibiotic hematotoxicity is best viewed as a biologically informative signal that can guide mechanism-based repurposing and combination strategies, but clinical development requires rigorous pharmacokinetic/pharmacodynamic validation, normal hematopoietic comparators, and biomarker-driven patient selection.

## Introduction

Antibiotics are among the most transformative advances in modern medicine, dramatically reducing mortality from bacterial infections and reshaping clinical care [1]. Although clinically used antibiotics were historically selected for favorable efficacy-versus-toxicity profiles [2], growing evidence shows that many interact with mammalian cellular targets and pathways, producing off-target effects that extend beyond antimicrobial activity [1]. A major mechanism is unintended inhibition of mitochondrial ribosomes by several ribosome-targeting antibiotics, reflecting the structural homology between bacterial ribosomes and mitoribosomes; however, non-ribosomal antibiotic classes can also disrupt core eukaryotic processes, including mitochondrial function, autophagy, apoptosis, and DNA replication [1, 3].

These off-target effects are particularly relevant in the hematopoietic system, where rapid turnover, lineage-specific differentiation, and high metabolic demand render marrow cells vulnerable to mitochondrial, oxidative, and cell-cycle stress [4, 5]. In parallel, immune-mediated mechanisms can selectively destroy erythrocytes, leukocytes, or platelets when drugs or metabolites act as haptens or trigger autoantibody formation [6]. Clinically, such toxicities are usually treated as adverse effects requiring monitoring, discontinuation, or dose adjustment. Yet they also raise a therapeutic question: if antibiotics can perturb normal hematopoietic proliferation and survival, can any of those perturbations be redirected against malignant blood cells? This question is especially relevant in leukemia, where malignant blasts and leukemia stem/progenitor populations display altered mitochondrial metabolism, redox buffering, autophagy, apoptotic priming, ribosome biogenesis, and microenvironment-mediated survival programs [7–13]. This perspective aligns with growing interest in antibiotic repurposing in oncology [14, 15].

**Fig. 1:**
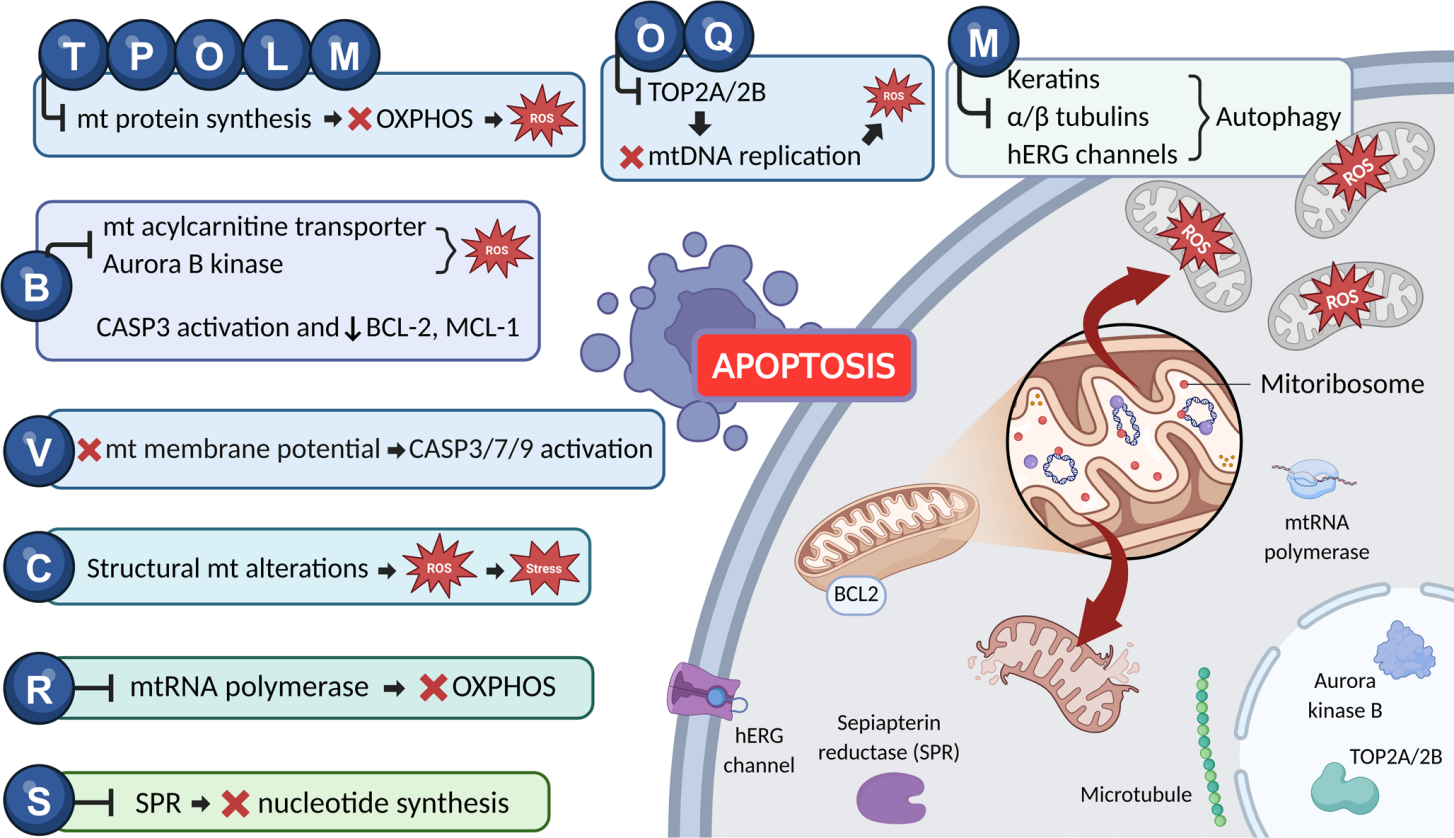
Overview of documented off-target effects of antibiotics in eukaryotic cells and their convergence on hematologic toxicity and leukemia-relevant stress pathways. Most effects converge on mitochondrial (mt) dysfunction, ROS production, altered autophagy/lysosomal flux, DNA or topoisomerase stress, and apoptosis. T = tetracyclines, P = phenicols, O = oxazolidinones, L = lincosamides (clindamycin), M = macrolides, B = beta-lactams, V = vancomycin, C = colistin, Q = fluoroquinolones, R = rifamycins, S = sulfonamides. The translational framework linking these effects to marrow toxicity and leukemia vulnerabilities is summarized in Table 5

In this narrative review, published evidence is synthesized on (i) antibiotic off-target effects in eukaryotic cells, (ii) antibiotic-induced hematologic toxicity, and (iii) cytotoxic and chemosensitizing effects of antibiotics in leukemia models. Relevant studies were identified through targeted PubMed and Scopus searches, with additional articles retrieved by screening reference lists. Mechanistic off-target effects are first summarized by antibiotic class (Table 1; Fig. 1). The revised core of the review then integrates hematologic toxicity with leukemia biology, using antibiotic-associated cytopenias as a window into vulnerable processes rather than as direct evidence of therapeutic selectivity (Table 2). Finally, preclinical antileukemic data are interpreted through this mechanism-based framework (Tables 3 and 4), and the translational barriers are considered explicitly. The aim is to reframe antibiotic hematotoxicity as a hypothesis-generating source of biologic insight while avoiding overinterpretation of predominantly in vitro evidence.

## Off-target effects of antibiotics in eukaryotic cells

Antibiotic off-target effects in eukaryotic cells are class-dependent and stem from structural or functional overlap between bacterial targets and mammalian pathways. Across classes, recurrent consequences include mitochondrial dysfunction, oxidative stress, altered autophagy, dysregulation of apoptosis, and impaired proliferation (Table 1; Fig. 1).

**Table 1:** Antibiotic mode of action against bacterial targets and off-target effects in eukaryotic cells

Type	Antibiotic Class	Representatives	Mechanism of action on bacterial cells	Off-target effects on eukaryotic cells
Ribosome inhibitors	Tetracyclines	Tigecycline, Tetracycline, Doxycycline, Medocycline, Methacycline, Minocycline, Oxycycline	Inhibit protein synthesis by binding to 30 S ribosomal subunit, preventing aminoacyl-tRNA attachment to the mRNA-ribosome complex [20]	Disrupt mitochondrial protein translation by preventing the accommodation of the incoming A-site tRNA and directly blocking the PTC on the human 55 S mitoribosome [20]
	Oxazolidinones	Linezolid, Tedizolid, Sutezolid, Eperezolid	Inhibit protein synthesis by binding to the 23 S rRNA of the 50 S ribosomal subunit, disrupting the assembly of 70 S initiation complex [25]	Disrupt mitochondrial protein translation by binding to the 16 S rRNA of the mitochondrial 28 S subunit [28, 31]
	Macrolides	Erythromycin, Clarithromycin, Azithromycin, Roxithromycin, Rokitamycin	Inhibit protein synthesis by binding to the 23 S rRNA of the 50 S ribosomal subunit, blocking the PTC and preventing polymerization of specific amino acid sequences [16, 35]	Modulate mitochondrial protein translation [35] Inhibit autophagy by disrupting cytoskeletal protein dynamics [42]
	Lincosamides	Lincomycin, Clindamycin, Pirlimycin	Modulate protein synthesis by binding to the 23 S rRNA of the 50 S ribosomal subunit, blocking the PTC and preventing peptide bond formation [214, 215]	Inhibit mitochondrial protein translation by binding at site corresponding to peptidyl-transferase center [214]
	Streptogramins	Streptogramin A, Streptogramin B	Streptogramin A binds PTC, enhancing Streptogramin B binding to adjacent NPET. Both target 23 S rRNA of 50 S subunit, blocking PTC and preventing peptide bond formation [16]	No reported streptogramin effects on eukaryotic cells
	Aminoglycosides	Streptomycin, Gentamicin, Kanamycin, Tobramycin	Alter protein synthesis by interacting with 30 S ribosomal subunit [216]	Modulate mitochondrial protein synthesis through a decrease in mRNA translation fidelity, which is caused by an inhibition of ribosome recycling via interactions with helices H44/H69 [216] Impaired cellular respiration and energy metabolism caused by hampering production of COX subunit I and II [1] Reduction of respiration rate, increase of NADH and ROS production and inhibition of mitochondrial complexes II and III due to aminoglycoside-induced loss of mitochondrial membrane potential [49, 217–220] Oxidative stress due to ROS over-production [1] Induces mitophagy through LC3-B increase [221]
RNA polymerase inhibitors	Rifamycins	Rifamycin	Disrupt transcription by inhibiting DNA-dependent RNA polymerase (RNAP) by either physically blocking RNA synthesis or by reducing RNAP’s affinity for short RNA sequences [222]	Disrupt transcription by inhibiting RNA polymerase II [222]
DNA replication inhibitors	Fluoroquinolones	Ciprofloxacin, Levofloxacin, Pefloxacin, Moxifloxacin, Lomefloxacin, Norfloxacin	Disrupt DNA replication by inhibiting DNA gyrase and topoisomerase IV, leading to unresolved double-strand DNA breaks [223]	Disrupt mtDNA replication by inhibiting mitochondrial topoisomerases I/II and DNA polymerase [60] Induce mitochondrial dysfunction and oxidative damage by disrupting the mitochondrial electron transport chain function leading to ROS over-production [49]
Inhibitors of cell wall biosynthesis	β-lactams	Penicillin, Ampicillin, Piperacillin, Amoxicillin, Ceftriaxone	Inhibit cell wall synthesis by interacting with penicillin-binding proteins (PBPs) [47]	Induce mitochondrial dysfunction and oxidative stress by increasing accumulation of mitochondrial superoxide and intracellular ROS [49, 50] Disrupt mitochondrial function by: ● Inhibiting the activity of mitochondrial complexes I, II, III and IV [49] ● Inducing apoptosis by increasing caspase-3 and inhibiting anti-apoptotic proteins Bcl-2 and Mcl-1 [50] ● Binding to the mitochondrial carnitine/acylcarnitine transporter [48]
	Glycopeptides	Vancomycin	Inhibit cell wall synthesis by blocking peptidoglycan tetrapeptide formation in Gram-positive bacteria [224]	Block autophagy and induce IL-1β release in macrophages [224]
	Sulfonamides	Sulfamethoxazole	Inhibit dihydropteroate synthase, blocking bacterial folic acid synthesis [70]	Inhibits sepiapterin reductase, disrupting tetrahydrobiopterin biosynthesis, affecting neurotransmitter synthesis, immune response and endothelial function [70]
	Polymyxins	Polymyxin B, Colistin	Membrane disruption via electrostatic interaction Phospholipid depletion ROS generation NDH-2/TCA cycle inhibition [225]	Induce apoptosis through the activation of caspase-3/8/9 pathways [226] Disruption of mitochondrial function leading to loss of membrane potential, ROS generation, decreased respiratory oxidoreductase activity [227] Inhibit respiratory enzymes, including NDH-2 activity and TCA cycle [225]

### Ribosome-targeting antibiotics

Mitochondrial susceptibility to antibiotic toxicity reflects the endosymbiotic origin of mitochondria and the retention of a specialized translation apparatus for mitochondrial DNA (mtDNA)-encoded oxidative phosphorylation (OXPHOS) components [1, 3, 16]. Human mitoribosomes retain key bacterial-like features [including conserved rRNA elements, peptidyl transferase center (PTC) architecture, and nascent peptide exit tunnel organization], providing a structural basis for off-target inhibition by several bacterial ribosome inhibitors [3, 16]. Structural evidence of mitoribosomal binding has recently emerged for selected classes, but functional evidence for mitochondrial translation inhibition has long preceded these cryo-electron microscopy (cryo-EM) data [1, 3, 17].

A shared downstream consequence is repression of mitochondrial translation, leading to mitonuclear protein imbalance (discordance between mtDNA-encoded and nuclear-encoded protein synthesis), impaired mitochondrial proteostasis, and progressive OXPHOS dysfunction [18] (Fig. 1). Selective depletion of mitochondrially encoded respiratory-chain subunits reduces electron transport efficiency, lowers NAD regeneration, perturbs the NAD+/NADH ratio, and disrupts energy-dependent cellular processes, including glycolysis and cytokine production [19] (Fig. 1). Antibiotic-induced mitochondrial dysfunction is also associated with reactive oxygen species (ROS) overproduction and broader metabolic stress, which can culminate in apoptosis or other forms of cell death [19] (Fig. 1).

#### Tetracyclines

Tetracyclines inhibit bacterial protein synthesis by binding to the 30 S ribosomal subunit and blocking aminoacyl-tRNA attachment; multiple tetracyclines also inhibit mitochondrial translation through the 55 S mitoribosome [1, 16]. Recent cryo-EM work showed that tigecycline binds to multiple sites within the human mitochondrial ribosomal PTC and globally suppresses mitochondrial protein synthesis in human cell lines [20].

#### Phenicols

Phenicols bind the 50 S PTC and block elongation in a context-dependent manner [21, 22]. Chloramphenicol has long been known to reversibly suppress mitochondrial protein synthesis in mammalian cells, and recent structural and mechanistic studies indicate a nascent peptide-dependent mode of inhibition analogous to that observed in bacterial ribosomes [17, 23]. This inhibition can reduce cytochrome c oxidase activity and ATP production and impair proliferation [24].

#### Oxazolidinones

Oxazolidinones (e.g., linezolid) bind the 50 S PTC and inhibit translation initiation in a context-dependent manner [25, 26]. Their mitochondrial toxicity is well documented in vitro and in vivo [27–30], and recent cryo-EM studies have confirmed direct mitoribosomal binding and nascent peptide-dependent inhibition of mitochondrial protein synthesis, resulting in reduced levels of respiratory-chain subunits, cytochrome c oxidase activity, and OXPHOS capacity [17, 28, 31].

#### Lincosamides

Lincosamides bind to 23 S rRNA within the 50 S PTC and inhibit peptide bond formation [16]. Recent studies show that clindamycin impairs mitochondrial membrane potential and OXPHOS and activates mitochondria-mediated cell death at clinically relevant concentrations [32, 33].

#### Macrolides

Macrolides inhibit bacterial protein synthesis by binding to the bacterial 50 S nascent peptide exit tunnel and blocking elongation in a sequence- and context-dependent manner [16, 34, 35]. Structural studies also show binding to the eukaryotic 80 S cytosolic ribosome, with sequence-selective stalling [36]. Although direct modern evidence for macrolide inhibition of mitochondrial translation remains limited (despite early erythromycin data in isolated cells) [37], multiple studies report macrolide-induced mitochondrial dysfunction, including increased ROS, reduced respiratory-chain activity, and impaired OXPHOS, which is mechanistically consistent with interference with mitochondrial translation [35, 38–40].

Macrolides also exert non-ribosomal effects in eukaryotic cells. Azithromycin potently inhibits autophagy by directly binding keratins (disrupting filament dynamics and lysosomal maturation) and α/β-tubulins (impairing microtubule-dependent lysosomal trafficking and autophagic flux) [41, 42]. Clarithromycin and erythromycin also block autophagic flux by inhibiting hERG1 potassium channels, which are constitutively expressed in primary AML and support leukemic progenitor proliferation via stromal interactions; hERG inhibition induces autophagy but subsequently blocks flux, promoting both autophagic and apoptotic cell death [43–45]. Roxithromycin can further suppress proliferation by modulating cytokinesis and cell-cycle progression [46].

### Beyond ribosome inhibition: Antibiotics with other modes of action

#### β-lactams

Several non-ribosomal-targeting antibiotics also exhibit significant eukaryotic off-target effects. β-lactams inhibit bacterial cell-wall synthesis through penicillin-binding proteins [1, 47], but several (including ampicillin, piperacillin, and selected cephalosporins) inhibit the mitochondrial carnitine/acylcarnitine transporter, potentially impairing fatty-acid β-oxidation and energy metabolism [48] (Fig. 1). Ampicillin inhibits electron transport chain complexes I–IV and induces oxidative damage [49], while piperacillin can promote mitochondria-mediated apoptosis with caspase-3 activation and reduced BCL-2/MCL-1 expression [50]. Increased ROS has also been reported with multiple β-lactams [49–51]. Beyond mitochondrial effects, ceftriaxone was recently identified as an Aurora B kinase inhibitor in lung cancer models (Fig. 1), indicating that some β-lactams may directly target eukaryotic kinases [52].

#### Glycopeptides

Glycopeptides block bacterial cell-wall synthesis by binding the C-terminal L-Lys–D-Ala–D-Ala motif of Lipid II [53] (Fig. 1). In mammalian cells, especially with high or prolonged exposure, vancomycin reduces viability, disrupts mitochondrial membrane potential, increases ROS, decreases ATP synthesis, and activates caspase-3/7- and caspase-9-dependent apoptosis [54–57]. Oxidative damage to cardiolipin, a key phospholipid of the inner mitochondrial membrane, may further aggravate mitochondrial dysfunction [57, 58].

#### Fluoroquinolones

Fluoroquinolones inhibit bacterial DNA gyrase and topoisomerase IV [1], but in mammalian cells they can inhibit TOP2A/TOP2B and disrupt mtDNA replication and transcription (Fig. 1), particularly through TOP2B, which regulates mtDNA supercoiling [1, 59, 60]. This can cause mtDNA depletion and OXPHOS impairment [60]. Additional mitochondrial targets have also been identified, including AIFM1 (Apoptosis-inducing factor mitochondrial 1) and IDH2 (Isocitrate dehydrogenase 2): binding to AIFM1 interferes with MIA40-dependent import and oxidative folding of nuclear-encoded mitochondrial proteins (contributing to downregulation of respiratory complexes I and IV), while IDH2 inhibition impairs NADPH generation and blocks metabolic compensation, amplifying mitochondrial toxicity [61–63].

#### Polymyxins

Polymyxins disrupt Gram-negative bacterial outer membranes [1], but colistin also damages mammalian mitochondria, increasing permeability, reducing enzyme activity, impairing ATP synthesis, and inducing mitochondrial apoptosis in experimental models [64].

#### Rifamycins

Rifamycins inhibit bacterial RNA polymerase [65] and can also inhibit mitochondrial RNA polymerase and mitochondrial RNA synthesis, with downstream effects including altered mitochondrial mass, ROS overproduction, electron transport chain impairment, ATP depletion, and apoptosis [66–68] (Fig. 1).

#### Sulfonamides

Sulfonamides (e.g., sulfamethoxazole) exert antibacterial activity by competitively inhibiting bacterial dihydropteroate synthase in the folate biosynthesis pathway. In the trimethoprim-sulfamethoxazole combination (TMP-SMX), trimethoprim inhibits the downstream enzyme dihydrofolate reductase, producing a sequential blockade of tetrahydrofolate biosynthesis and consequent impairment of folate-dependent thymidine synthesis [69]. Emerging evidence also indicates eukaryotic off-target effects: although mammalian cells lack dihydropteroate synthase, sulfonamides can inhibit sepiapterin reductase, potentially disrupting tetrahydrobiopterin-dependent processes involved in monoamine biosynthesis, immune modulation, and endothelial function [70, 71]. In addition, a reactive sulfamethoxazole metabolite induces apoptosis in CD8 + T cells (phosphatidylserine externalization and internucleosomal DNA fragmentation), providing a mechanistic basis for sulfonamide-associated hypersensitivity [72–74].

## Antibiotic hematotoxicity as a window into leukemia vulnerabilities

The conceptual bridge between antibiotic hematologic toxicity and antileukemic repurposing is not that any cytopenia predicts antileukemic efficacy. Rather, antibiotic-induced cytopenias identify cellular processes with limited tolerance in hematopoietic tissues. Some of these processes - mitochondrial translation, oxidative phosphorylation (OXPHOS), redox control, autophagy, DNA replication, ribosome biogenesis, and apoptotic thresholding - are also central to leukemia cell survival and therapy resistance [7–13]. Table 2 is therefore used as a clinical safety context: cytopenias driven by direct marrow stress are mechanistically informative, whereas immune-mediated cytopenias mainly define toxicity liabilities and do not, by themselves, imply leukemia selectivity. The key translational question is whether a given antibiotic perturbs a vulnerability that is more limiting in leukemic cells than in normal hematopoietic stem and progenitor cells.

**Table 2:** Antibiotic-associated leukopenia and neutropenia/agranulocytosis based on clinical product information

Category	Antibiotic	Leukopenia	Neutropenia ± Agranulocytosis
Penicillins	Penicillin G benzathine	Very rare	Very rare
	Amoxicillin	Very rare	Very rare
	Amoxicillin–clavulanate	Rare	Rare
	Ampicillin	Uncommon	Uncommon
	Ampicillin–sulbactam	Uncommon	Uncommon
	Flucloxacillin	Very rare	Very rare
	Piperacillin–tazobactam	Uncommon	Rare
Cephalosporins	Cefadroxil	Rare	Rare
	Cefaclor	Rare	Rare
	Ceftriaxone	Common	Common
	Cefotaxime	Uncommon	Uncommon
	Cefepime	Rare	Rare
	Cefuroxime	Uncommon	Uncommon
	Cefazolin	Rare	Rare
	Ceftaroline	Uncommon	Uncommon
	Ceftazidime	Uncommon	Uncommon
	Ceftazidime–avibactam	Uncommon	Uncommon
Carbapenems	Meropenem	Uncommon	Uncommon
	Imipenem–cilastatin	Uncommon	Uncommon
	Ertapenem	Rare	Uncommon
Monobactams	Aztreonam	Very rare	Rare
Macrolides	Azithromycin	Uncommon	Uncommon
	Clarithromycin	Uncommon	Uncommon
Lincosamides	Clindamycin	Common	Common
	Lincomycin	Very rare	Very rare
Streptogramins	Quinupristin and Dalfopristin (Pristinamycin)	Rare	Rare
Tetracyclines	Doxycycline	Rare	Rare
	Minocycline	Rare	Rare
	Tigecycline	Very rare	Very rare
Glycopeptides	Vancomycin	Rare	Rare
	Teicoplanin	Uncommon	Very rare
	Telavancin	Uncommon	Uncommon
Lipopeptides	Daptomycin	Very rare	Very rare
Oxazolidinones	Linezolid	Uncommon	Uncommon
Fluoroquinolones	Ciprofloxacin	Rare	Rare
	Levofloxacin	Uncommon	Rare
	Norfloxacin	Rare	Rare
Sulfonamides	Trimethoprim–sulfamethoxazole	Common	Common
Nitroimidazoles	Metronidazole	Very rare	Very rare
Nitrofurans	Nitrofurantoin	Very rare	Very rare
Rifamycins	Rifampicin	Uncommon	Very rare
Phenicols	Chloramphenicol	Uncommon	Uncommon
Phosphonic acid derivatives	Fosfomycin	Very rare	Very rare
Polymyxins	Polymyxin B	Very rare	Very rare
	Colistin	Uncommon	Uncommon
Aminoglycosides	Amikacin	Rare	Rare
	Gentamicin	Very rare	Very rare

Frequency descriptors used: Common (≥ 1/100 to < 1/10), Uncommon (≥ 1/1,000 to < 1/100), Rare (≥ 1/10,000 to < 1/1,000), Very rare (< 1/10,000). Frequencies were extracted from Summary of Product Characteristics/product-information sources available through the electronic Medicines Compendium (https://www.medicines.org.uk/emc/; last accessed: June 11, 2026). CTCAE v6.0 was used only as a standard adverse-event terminology and grading framework for leukopenia, neutropenia and ageb 2ranulocytosis, not as a source of frequency estimates. Because reported frequencies may vary by formulation, indication, dose, exposure duration and population, the table should be interpreted as a clinical safety-context map rather than as a pooled incidence analysis

### Direct myelosuppression and immune cytopenia are biologically distinct

Antibiotic-associated hematologic toxicity reflects at least two overlapping mechanisms: direct marrow/progenitor stress and immune-mediated peripheral destruction (Table 2) [75–81]. Direct myelosuppression includes mitochondrial injury, inhibition of mitochondrial or cytosolic protein synthesis, DNA damage, folate-pathway interference, oxidative stress, and impaired lineage maturation. Immune-mediated toxicity includes hapten-dependent hemolysis, drug-dependent antiplatelet or antineutrophil antibodies, and hypersensitivity-associated eosinophilia [6, 75, 78, 82–86, 93–98, 103–156]. This class-spanning evidence covers tetracycline-associated eosinophilia, thrombocytopenia, and rare marrow events; macrolide-associated cytopenias and lymphocyte apoptosis; oxazolidinone and lincosamide hematotoxicity; beta-lactam, carbapenem, glycopeptide, fluoroquinolone, polymyxin, rifamycin, and TMP-SMX-associated cytopenias; and both direct and immune-mediated mechanisms [82–156]. This distinction is central to the present review because direct progenitor toxicity can plausibly share biology with leukemia-cell killing, whereas immune cytopenias are clinically important safety signals but provide a weaker rationale for repurposing unless supported by direct tumor-cell data.

The clinical manifestations also differ in their mechanistic informativeness. Linezolid-associated thrombocytopenia and anemia, chloramphenicol-associated reversible marrow suppression, and trimethoprim-associated neutropenia each point toward direct effects on hematopoietic production [4, 23, 30, 89–91, 99–101, 146]. In contrast, ceftriaxone-associated immune hemolysis, vancomycin-induced neutropenia, quinolone-associated immune thrombocytopenia, and rifampicin-dependent hemolysis mainly reflect drug-dependent immune recognition rather than selective pressure on malignant hematopoietic cells [83, 111, 122–132, 135]. Thus, the toxicity signal must be mechanistically deconvoluted before it is used to support repurposing.

### Mitochondrial translation and OXPHOS dependence: the strongest mechanistic bridge

Mitochondrial translation inhibition provides the most coherent link between hematotoxicity and antileukemic activity. Normal marrow cells require mitochondrial function during lineage commitment, megakaryocytic maturation, erythropoiesis, immune activation, T-cell function, and stress hematopoiesis [4, 5, 19, 101, 158–160]. Antibiotics that inhibit mitoribosomes can therefore produce cytopenias when exposure exceeds lineage-specific tolerance. Chloramphenicol established this principle historically through reversible, dose-related marrow suppression associated with mitochondrial injury in erythroid, myeloid, and megakaryocytic lineages [23, 89–91]. Linezolid and related oxazolidinones provide a contemporary example, with mitochondrial dysfunction contributing to impaired megakaryocyte differentiation and platelet production [4, 30, 99–101].

The same axis is attractive in AML and other leukemias because leukemic blasts and leukemic stem/progenitor compartments can show heightened mitochondrial biogenesis, OXPHOS dependence, amino-acid-fueled respiration, and adaptive mitochondrial metabolism under therapeutic stress [7–10, 161–164, 168, 187]. Tigecycline is the clearest proof-of-concept: it inhibits mitochondrial translation, produces mitonuclear protein imbalance, and selectively impairs AML stem/progenitor survival in preclinical models [20, 161–165]. However, the therapeutic window is narrow and context-dependent. A compound that injures leukemic mitochondria may also injure megakaryocytes, erythroid precursors, activated T cells, or normal CD34 + cells. Therefore, future studies should report normal hematopoietic comparators, lineage-specific toxicity, mitochondrial pharmacodynamic markers, and reversibility rather than leukemia-cell IC50 values alone.

### ROS, DNA damage, and apoptotic priming

Reactive oxygen species (ROS) generation is another recurring antibiotic off-target effect, but it is mechanistically double-edged. Excess ROS can damage mitochondrial membranes, proteins, and DNA, induce apoptosis, and impair marrow progenitors [49–51, 54–64]. In leukemia, redox biology is heterogeneous: bulk blasts may show oxidative stress, whereas leukemia stem cells often maintain low ROS states and depend on mitochondrial integrity, BCL-2-regulated survival, autophagy, and antioxidant buffering [7, 11]. Antibiotic-induced ROS may therefore be exploitable only when leukemic cells are closer to an oxidative death threshold than normal progenitors, or when ROS cooperates with DNA damage, topoisomerase stress, mitochondrial depolarization, or impaired anti-apoptotic buffering [175, 176, 192, 196–203].

This framework clarifies why ROS-generating antibiotics should not be advanced solely on the basis of in vitro cytotoxicity. ROS production is also a major mechanism of nephrotoxicity, neurotoxicity, cardiotoxicity, and marrow injury for several antibiotic classes [49–64]. Mechanistic studies should therefore include antioxidant-rescue experiments, mitochondrial membrane-potential assays, caspase dependence, DNA-damage markers, and direct comparison with normal hematopoietic progenitors. Without these controls, ROS-associated leukemia-cell death may simply reflect nonspecific chemical stress.

### Autophagy and lysosomal flux: vulnerability, resistance mechanism, and toxicity amplifier

Autophagy is a second major point of convergence between antibiotic off-target effects, hematopoiesis, and leukemia. In established AML, autophagy can support leukemia-cell survival, contribute to stromal protection, and mediate resistance to chemotherapy or targeted agents [7, 12]. This makes macrolide-mediated blockade of autophagic flux biologically relevant, particularly in marrow-like coculture systems where stromal support protects leukemic cells from cytotoxic therapy [41–45]. Clarithromycin-induced inhibition of late-stage autophagy and hERG1-dependent effects in AML and CML models therefore provides a mechanistic rationale for synergy with cytarabine, dasatinib, and other antileukemic agents [45, 181–183].

At the same time, autophagy has context-dependent effects in normal and malignant hematopoiesis. It can preserve organelle quality, support stress adaptation, or contribute to cell death depending on cell state, genetic context, nutrient availability, and microenvironmental oxygen tension [7, 12]. Consequently, autophagy inhibition should be interpreted as a combination strategy rather than a universally selective antileukemic mechanism. Studies should measure flux rather than static LC3 or p62 levels alone, should test stromal and hypoxic conditions, and should evaluate whether autophagy blockade sensitizes leukemic cells without unacceptable injury to normal hematopoietic or immune compartments.

### Ribosome biogenesis, topoisomerase stress, folate stress, and other proliferative vulnerabilities

Several non-mitoribosomal antibiotic effects intersect with proliferative vulnerabilities in leukemia. Fluoroquinolones and selected derivatives can inhibit mammalian topoisomerases and disrupt mtDNA replication, leading to replication stress, S-phase arrest, and apoptosis [59–62, 188–195]. A linezolid-specific metabolite can inhibit TOP2A/TOP2B, offering a plausible explanation for marrow toxicity that extends beyond mitoribosomal inhibition [30]. Trimethoprim produces folate-pathway stress and can amplify methotrexate exposure in children with ALL, a clinically important interaction that illustrates how anti-infective drugs can alter antimetabolite pharmacology even when direct antileukemic cytotoxicity is weak [207, 208].

Ribosome biogenesis is also a process already implicated in malignant hematopoiesis. Although most ribosome-targeting antibiotics discussed here act on bacterial ribosomes or mitoribosomes rather than directly targeting nucleolar ribosome biogenesis, translational stress, mitonuclear imbalance, and nucleolar stress converge on related growth-control pathways. Recent AML work has emphasized that ribosome-biogenesis activity can mark aggressive disease biology and leukemic progenitor states [13]. This strengthens the rationale for examining whether antibiotic-induced translation stress interacts with ribosome-biogenesis programs, MYC-driven growth, p53 status, and stress-response signaling in leukemia.

## Mechanism-informed evidence for antileukemic activity by antibiotic class

Preclinical studies show that several antibiotic classes can kill leukemia cells or enhance standard therapies (Table 3). The evidentiary strength varies substantially, however, and should be interpreted through the mechanisms outlined above rather than by class membership alone.

**Table 3:** Cytotoxic effects of antibiotics on diverse leukemia cell lines

Category	Antibiotic	Main Findings	References
Tetracyclines	Tigecycline	Potent dose-dependent cytotoxicity against the AML TEX and M9-ENL1, the T-ALL Jurkat, the ALL CCRF-CEM, DND-41 and MOLT-16 and the CML K562 and KBM5 cell lines Enhances chemotherapy efficacy when combined with doxorubicin, vincristine, or homoharringtonine; re-sensitizes resistant CML stem cells to imatinib	[20, 157, 161, 162, 164–169]
	Doxycycline	Concentration- and time-dependent growth inhibition/apoptosis against T-ALL Jurkat and CCRF-CEM, AML/APL HL-60, KG1a and CML K562 models; reduced relapse in murine T-cell leukemia when combined with adriamycin or cytarabine	[157, 170–174]
	Minocycline	Potent cytotoxic effects against the AML HL-60, the T-ALL Jurkat and the CML K562 cell lines Selectively induces apoptosis in T-ALL Jurkat cells	[172, 175, 176]
Phenicols	Chloramphenicol	Dose-dependent cytotoxicity against the CML K562 cell line Reduced white cell and blast count in a CML patient	[178, 179]
Macrolides	Clarithromycin	Dose-dependent cytotoxicity against the AML FLG 29.1 and HL-60, B-ALL REH and the CML K562 and K562 Dox-resistant cell lines Synergistic effects with cytarabine and dasatinib Potentiates TKI efficacy in TKI-resistant CML patients	[45, 46, 181–183]
	Erythromycin	Dose-dependent cytotoxicity against the ALL REH and 697 and the AML HL-60 cell lines Synergistic effects with prednisone Suppressed leukemic cell engraftment in bone marrow	[45, 182]
	Azithromycin	Moderate cytotoxic effects against the AML HL-60, the T-ALL Jurkat and the CML K562 cell lines	[42, 184–186]
	Rokitamycin	Potent cytotoxicity against the AML HL-60 and T-ALL Jurkat cell lines	[182]
	Roxithromycin, Josamycin, Leucomycin	Moderate and dose-dependent cytotoxicity against the AML HL-60 cell line	[46, 182]
Oxazolidinones	Linezolid	Dose-dependent cytotoxicity against the AML TEX, HL-60 and THP-1 cell lines	[30, 161]
	Tedizolid	Dose-dependent cytotoxicity against the AML HL-60, THP-1 and MOLM-13 cell lines Overcomes venetoclax resistance in MOLM-13; triplet combination with venetoclax and azacitidine showed enhanced cytotoxic activity	[29, 187]
Fluoroquinolones	Ciprofloxacin	Dose-dependent cytotoxicity with enhanced activity in novel derivatives against the CML K562 and the AML HL-60 and KG-1 cell lines	[188, 190, 191]
	Levofloxacin	Levofloxacin-based compounds with potent cytotoxic activity against the L-SR cell line	[192]
	Pefloxacin, Lomefloxacin, Norfloxacin, Moxifloxacin	Dose-dependent activity against the AML HL-60, the monocytic leukemia THP-1 and the T-ALL Jurkat cell lines Moxifloxacin enhances the anti-proliferative activity of etoposide	[188, 193–195]
Beta-lactams	Penicillin G	Dose- and time-dependent cytotoxicity against the CML K562 cell line	[196]
	N-thiolated β-lactams	Cytotoxicity through apoptosis induction against the T-ALL Jurkat cell line	[197–199]
Polymyxins	Polymyxin B and Colistin	Cytotoxicity against the ALL REH cell line Colistin induces necrotic cell death in the monocytic leukemia THP-1, erythroleukemia TF-1 and the AML MV-4-11 cell lines	[200–203]
Rifamycins	Rifampicin	Increased accumulation of vinblastine in multidrug-resistant CML K562 cells No reported effect in drug-sensitive cells	[204, 205]
Glycopeptides	Teicoplanin	Dose-dependent cytotoxicity against the T-ALL Jurkat cell line at concentrations more than twice those used clinically	[206]
Folate pathway inhibitors	Trimethoprim	Inhibited DNA incorporation by 50% in the murine L1210 leukemia cell line	[207, 208]

### Mitochondrial translation inhibitors: tetracyclines, phenicols, and oxazolidinones

Tetracyclines, especially tigecycline, remain the most convincing class for antileukemic repurposing. Tigecycline emerged as the most active compound in a 312-compound AML screen and has shown cytotoxicity across AML, ALL, and CML models, including patient-derived leukemic cells [20, 161–166]. Its combination activity with doxorubicin, vincristine, homoharringtonine, and imatinib-resistant CML stem-cell models reinforces the concept that mitochondrial translation inhibition may lower the threshold for conventional or targeted therapy [162, 167, 168]. Importantly, the negative phase I tigecycline trial in relapsed/refractory AML should not be dismissed as biologic refutation: the trial demonstrated that short half-life and insufficient sustained exposure prevented mitochondrial-translation inhibition at clinically achievable levels [169]. It therefore defines the central translational problem - exposure and pharmacodynamic target engagement - rather than eliminating the mechanism.

Doxycycline and minocycline provide supporting but less mature evidence. Doxycycline reduced relapse in murine T-cell leukemia combination models and inhibits growth, migration, and invasion in several leukemia cell lines [170–174]. Minocycline induces apoptosis through oxidative stress, mitochondrial depolarization, caspase activation, apoptosis-inducing factor signaling, DNA damage, and Bcl-xL degradation in Jurkat, HL-60, and K562 models [172, 175, 176]. These findings are mechanistically plausible but still require systematic comparison with normal hematopoietic progenitors and clinically relevant exposure ranges.

Phenicols and oxazolidinones reinforce the same biology but with important safety caveats. Chloramphenicol can produce reversible, dose-dependent cytotoxicity in K562 cells and has a historical CML case report suggesting clinical activity [177–179], but its known marrow toxicity and idiosyncratic aplastic-anemia risk limit therapeutic enthusiasm [87–92]. Linezolid and tedizolid show AML cytotoxicity signatures consistent with mitochondrial translation inhibition, and tedizolid can overcome venetoclax resistance in MOLM-13 cells when combined with venetoclax and azacitidine [29, 30, 161, 187]. The translational lesson is not that oxazolidinones should be broadly used as antileukemic drugs, but that mitoribosomal inhibition, integrated stress response activation, and glycolytic suppression may be rational drug-development targets.

### Autophagy-modulating macrolides

Macrolides are notable because their antileukemic effects are not explained solely by mitochondrial dysfunction. Clarithromycin, erythromycin, azithromycin, rokitamycin, roxithromycin, josamycin, and leucomycin show variable cytotoxicity in AML, ALL, and CML models [42, 45, 46, 180–186]. Clarithromycin is the most developed example: it inhibits leukemic cell lines, reduces AML burden in vivo, synergizes with cytarabine in pediatric AML samples and xenografts, and shows enhanced activity in mesenchymal stromal cell-supported cultures that approximate marrow-mediated chemoprotection [45, 181–183]. In CML, clarithromycin enhances dasatinib activity and restores sensitivity in resistant models, with clinical observations of improved molecular responses in TKI-resistant patients [181, 183].

This macrolide literature should be interpreted critically. Clarithromycin has clinically important CYP3A4- and transporter-related interaction potential and can therefore alter exposure to co-administered antileukemic agents, particularly TKIs and other CYP3A4 substrates [180, 183]. Therefore, apparent synergy may reflect a mixture of direct leukemia-cell autophagy/lysosomal effects, altered drug transport, microenvironmental disruption, and pharmacokinetic boosting. Future studies should separate these mechanisms by measuring intracellular drug accumulation, autophagy flux, hERG1 dependence, stromal protection, and leukemia-cell killing at concentrations achievable without unacceptable QT, drug-interaction, or antimicrobial-stewardship concerns.

### DNA/topoisomerase, folate, epigenetic, and cell-cycle stressors

Fluoroquinolones, selected beta-lactams, polymyxins, rifamycins, glycopeptides, and TMP-SMX occupy a lower level of evidence. Fluoroquinolone activity in leukemia models is long-standing, and newer derivatives can inhibit topoisomerase II beta, trigger replication stress, and induce caspase-mediated apoptosis [188–195]. This provides a mechanistic link to leukemia-cell proliferation, but it also raises genotoxicity and normal-progenitor safety concerns. Penicillin G and N-thiolated beta-lactams can induce cytotoxicity in K562 or Jurkat models, with reported STAT5/p53 or DNA-damage/apoptosis signals [196–199]; however, evidence remains sparse and mostly in vitro.

Polymyxin B and colistin are cytotoxic to REH, THP-1, TF-1, and MV-4-11 leukemia cells, and colistin/dextrin conjugation may shift cell death toward caspase-dependent apoptosis [200–203]. The identification of LSD1/KDM1A inhibition as a potential polymyxin-related mechanism is intriguing because LSD1 inhibition can promote AML differentiation [201, 202]. Nevertheless, polymyxin nephrotoxicity, neurotoxicity, membrane activity, and the high concentrations often needed for tumor-cell effects remain major barriers. Rifampicin appears more relevant as a modulator of multidrug resistance through P-glycoprotein effects than as a direct antileukemic agent [204, 205]. Teicoplanin and trimethoprim provide limited signals only at high or pharmacologically interaction-prone exposures [206–208].

### Evidence hierarchy

A practical hierarchy emerges from the current evidence (Table 4). Tetracyclines have the strongest mechanistic and preclinical foundation but have already exposed the PK/PD barrier in clinical testing. Macrolides have meaningful combination and microenvironmental data but require careful separation of direct antileukemic effects from pharmacokinetic drug-interaction effects. Oxazolidinones are mechanistically interesting, particularly in venetoclax-resistant AML models, but their hematologic toxicity overlaps with the target tissue. Fluoroquinolone and polymyxin derivatives are discovery leads rather than ready repurposing candidates. Beta-lactams, glycopeptides, rifamycins, and TMP-SMX currently provide mechanistic clues or interaction signals rather than sufficient evidence for leukemia-directed clinical translation.

**Table 4:** The highest level of evidence for anti-leukemic activity by antibiotic class

Category	Level of Evidence	Main Findings
Tetracyclines	Clinical trial	Despite potent cytotoxicity both in vitro and in vivo, a phase I trial of tigecycline in 27 patients with relapsed/refractory AML failed to induce clinical responses [169]
Phenicols	In vitro	Reversible, dose-dependent cytotoxicity against CML K562 cell line associated with cell cycle arrest and caspase-dependent apoptosis [179]
Macrolides	Clinical report	Clarithromycin potentiates TKI efficacy in TKI-resistant CML patients [183]
Oxazolidinones	In vivo	Linezolid has demonstrated in vitro cytotoxic activity against the AML TEX and HL-60 and the monocytic THP-1 cell lines [30, 161] Combination of tedizolid, venetoclax and azacitidine demonstrated enhanced cytotoxic activity both in vitro and in vivo [187]
Fluoroquinolones	In vitro	Ciprofloxacin, pefloxacin, lomefloxacin and norfloxacin exhibit dose-dependent inhibitory activity against the AML HL-60 cell line, with ciprofloxacin and pefloxacin additionally showing cytotoxic effects against the CML K562 cell line [188, 189, 193, 195]
Beta-lactams	In vitro	Dose- and time-dependent cytotoxicity of Penicillin G against the CML K562 cells [196]
Polymyxins	In vitro	Cytotoxic effects against REH ALL, THP-1 monocytic leukemia, TF-1 erythroleukemia and MV-4-11 AML cells [201, 203]
Rifamycins	In vitro	Rifampicin markedly increased vinblastine accumulation in multidrug-resistant CML K562 cells by modulating P-gp transporter activity [204]
Glycopeptides	In vitro	Teicoplanin exerts dose-dependent cytotoxicity on T-ALL Jurkat cell line [206]
TMP-SMX	Clinical pharmacokinetic interaction / limited in vitro signal	Trimethoprim inhibits DNA precursor incorporation in murine L1210 leukemia cells, while TMP-SMX increases systemic methotrexate exposure in children with ALL; this supports caution around drug interactions rather than direct clinical antileukemic efficacy [207, 208]

## Translational challenges and resistance considerations

### Biological relevance of in vitro findings

The main limitation of the field is that leukemia-cell cytotoxicity is often reported at concentrations, exposure durations, oxygen tensions, or culture conditions that may not reflect bone marrow pharmacology [169, 209]. Cell-line death in normoxic monoculture is particularly vulnerable to overinterpretation. Leukemia cells in vivo are shaped by hypoxia, nutrient limitation, stromal contact, cytokines, immune cells, drug gradients, and prior therapy. Therefore, clinically relevant evaluation should include patient-derived samples, normal CD34 + hematopoietic comparators, stromal cocultures, hypoxic or nutrient-stressed conditions, xenograft or organoid-like systems where appropriate, and pharmacodynamic markers demonstrating that the proposed target is actually engaged.

### Therapeutic window and in vivo off-target effects

A therapeutic window cannot be inferred from leukemia-cell killing alone. The same pathways that create antileukemic opportunities - mitochondrial translation, OXPHOS, ROS handling, autophagy, DNA replication, and apoptosis - are also required for normal hematopoiesis, immune competence, gut and renal function, cardiac electrophysiology, and tissue repair. This is especially important in patients with leukemia, who already experience cytopenias, mucosal barrier injury, infection risk, and polypharmacy. Any repurposing strategy must therefore define whether the intended antileukemic exposure is compatible with marrow recovery, antimicrobial stewardship, microbiome preservation, and avoidance of overlapping myelosuppression with chemotherapy, venetoclax, hypomethylating agents, TKIs, or antifungals.

### Pharmacokinetic/pharmacodynamic constraints

The tigecycline AML trial is the clearest warning that strong in vitro activity may fail when sustained bone marrow exposure is inadequate [169]. Similar PK/PD questions apply to macrolides, oxazolidinones, fluoroquinolones, and polymyxins: plasma concentrations may not equal intracellular leukemic concentrations; protein binding, efflux transporters, organ toxicity, and dosing ceilings may prevent target engagement; and intermittent antimicrobial dosing may not reproduce the continuous exposure used in vitro. Future studies should therefore pair cytotoxicity assays with achievable unbound concentrations, intracellular accumulation, marrow penetration, exposure-time modeling, and pharmacodynamic readouts such as depletion of mitochondrially encoded OXPHOS proteins, oxygen-consumption suppression, integrated stress response activation, autophagy-flux blockade, or topoisomerase/DNA-damage markers.

### Resistance mechanisms and rational combinations

Resistance may arise through metabolic plasticity, increased mitochondrial biogenesis, glycolytic compensation, enhanced mitophagy/autophagy, antioxidant adaptation, drug efflux, altered apoptotic priming, target remodeling, or microenvironmental protection [7, 163, 181, 186, 204, 205]. These mechanisms argue for rational combinations rather than antibiotic monotherapy. Examples include mitochondrial translation inhibitors with chemotherapy or venetoclax-based regimens, macrolide-mediated autophagy blockade with cytarabine or TKIs, and efflux-modulating antibiotics with agents affected by transporter activity [45, 162, 167, 168, 181, 183, 187, 204]. However, combination strategies must distinguish true biologic synergy from pharmacokinetic boosting and must avoid combinations that simply intensify marrow suppression or infectious risk.

### Biomarker-driven development

The most realistic clinical path is biomarker-driven adjuvant development rather than broad antibiotic monotherapy. Candidate biomarkers include high mitochondrial mass or OXPHOS dependence, sensitivity to mitochondrial translation inhibition, BCL-2/OXPHOS dependence, venetoclax-resistance states with preserved mitochondrial vulnerability, hERG1 expression or autophagy-flux dependence, P-glycoprotein-mediated resistance, TOP2-related response markers, and stromal protection phenotypes. Early-phase trials should incorporate pharmacodynamic endpoints and normal hematopoietic monitoring as co-primary translational objectives rather than exploratory afterthoughts.

## Limitations and future directions

This review has several limitations. First, it is a narrative synthesis rather than a systematic review or meta-analysis; therefore, it should be interpreted as a mechanistic and translational framework rather than a quantitative estimate of effect size. Second, the evidence base is uneven across antibiotic classes. Tetracyclines, macrolides, and oxazolidinones have relatively mature mechanistic or preclinical datasets, whereas cephalosporins, carbapenems, lincosamides, glycopeptides, TMP-SMX, rifamycins, and polymyxins are supported by smaller, more heterogeneous, or less leukemia-specific data. Third, many studies rely on immortalized cell lines, supratherapeutic concentrations, short viability assays, and limited normal hematopoietic comparators. Fourth, hematologic toxicity is mechanistically heterogeneous; immune cytopenias and direct marrow suppression should not be conflated. Finally, clinical translation is constrained by pharmacokinetics, drug interactions, antimicrobial stewardship, microbiome effects, organ toxicities, and overlapping myelosuppression in already fragile patients with leukemia. Clinical signals reported for clarithromycin in indolent lymphomas and the broader literature linking antibiotic exposure with outcomes during immune-checkpoint inhibitor therapy illustrate that antimicrobial exposure can have oncologic consequences, but these observations should not be extrapolated directly to acute leukemia without disease-specific mechanistic and clinical testing [210–213].

Future work should prioritize experiments that directly address these limitations: (i) systematic screening across AML, ALL, and CML panels with paired normal CD34 + and lineage-committed progenitor controls; (ii) patient-derived and genetically annotated models, including venetoclax-resistant, TKI-resistant, high-OXPHOS, autophagy-dependent, and stromal-protected phenotypes; (iii) clinically realistic unbound drug concentrations and exposure-time modeling; (iv) pharmacodynamic confirmation of mitochondrial translation inhibition, OXPHOS suppression, ROS/DNA damage, autophagy-flux blockade, topoisomerase inhibition, or ribosome-biogenesis stress; (v) in vivo validation with marrow, immune, renal, hepatic, cardiac, and microbiome safety readouts; and (vi) rational combinations designed around mechanism rather than around antimicrobial class alone. In this setting, antibiotics may serve not only as repurposed agents but also as chemical probes that reveal druggable leukemia dependencies.

## Concluding remarks

The evidence synthesized in this review supports a biologically plausible but still immature progression from antibiotic off-target effects to hematologic toxicity and, in selected settings, antileukemic activity. Mitoribosomal inhibition, mitochondrial dysfunction, ROS generation, autophagy/lysosomal-flux modulation, apoptosis regulation, topoisomerase stress, folate stress, and ribosome-biogenesis-associated growth programs provide mechanistic bridges between normal hematopoietic injury and leukemia-cell vulnerability (Table 5). The strongest translational rationale currently lies with mitochondrial translation inhibitors and autophagy-modulating macrolides used as rational adjuncts, not as empiric substitutes for established antileukemic therapy.

**Table 5:** Mechanism-to-vulnerability framework for interpreting antibiotic hematotoxicity and antileukemic repurposing

Antibiotic-linked biological activity	Hematologic toxicity signal	Leukemia-relevant vulnerability	Representative antibiotic classes	Key translational questions
Mitochondrial translation inhibition / OXPHOS suppression	Direct marrow suppression; impaired erythroid, myeloid, megakaryocytic or T-cell expansion	High mitochondrial mass, OXPHOS dependence, LSC metabolism, venetoclax- or chemotherapy-resistant states	Tetracyclines, phenicols, oxazolidinones, selected macrolides	Can clinically achievable marrow exposure suppress mitochondrial translation in blasts more than in normal CD34 + cells?
ROS generation, mitochondrial depolarization and apoptosis	Myelosuppression, lymphocyte apoptosis, lineage injury and organ toxicities	Oxidative death threshold, apoptotic priming, defective antioxidant buffering, mitochondrial fragility	Tetracyclines, beta-lactams, glycopeptides, fluoroquinolones, polymyxins	Is leukemia-cell death rescued by antioxidants or caspase blockade, and is the therapeutic index acceptable in normal progenitors?
Autophagy / lysosomal-flux blockade	Immune-cell and progenitor stress; possible amplification of toxicity under stress conditions	Cytoprotective autophagy, stromal chemoprotection, TKI/chemotherapy resistance, mitophagy dependence	Macrolides, selected glycopeptide/off-target autophagy modulators	Does true flux inhibition occur at achievable concentrations and sensitize leukemic cells without disabling normal hematopoietic recovery?
DNA replication, topoisomerase, folate and ribosome-biogenesis stress	Marrow suppression, neutropenia, genotoxicity or antimetabolite potentiation	Proliferative dependence, TOP2 vulnerability, nucleotide stress, growth-control and nucleolar/ribosome-biogenesis programs	Fluoroquinolones, linezolid metabolite, TMP-SMX/trimethoprim, selected beta-lactams	Is the effect direct, selective and biomarker-defined, or only a nonspecific antiproliferative/toxic signal?
Immune-mediated cytopenias	Drug-dependent hemolysis, thrombocytopenia, neutropenia, eosinophilia	Usually weak direct repurposing signal unless accompanied by leukemia-cell intrinsic effects	Beta-lactams, vancomycin, quinolones, rifampicin, TMP-SMX	Does the toxicity reflect immune destruction rather than targetable leukemia biology, and would it restrict safe combination therapy?

At the same time, antibiotic hematotoxicity should be interpreted cautiously. Cytopenia is not evidence of selective leukemia killing, and in vitro cytotoxicity is not evidence of clinical feasibility. The field will advance only if future studies define drug exposure, target engagement, biomarkers of response, resistance mechanisms, and normal hematopoietic safety with the same rigor used for conventional anticancer drug development. Viewed in this way, antibiotic-induced hematologic toxicity is not merely an adverse-effect category; it is a hypothesis-generating signal that can help identify vulnerabilities for mechanism-based leukemia therapy and future drug development.

## Data Availability

No datasets were generated or analysed during the current study.
